# Encephalocraniocutaneous lipomatosis: a rare and sporadic phakomatosis^؆^

**DOI:** 10.1016/j.abd.2025.501276

**Published:** 2026-01-19

**Authors:** Ana Clara Maia Palhano, Julia Maria de Oliveira Neumayer, Milene Tiburcio Narenti Ferradoza, Luciana Paula Samorano, Maria Cecilia Rivitti-Machado, Zilda Najjar Prado de Oliveira

**Affiliations:** Department of Dermatology, Faculty of Medicine, Hospital das Clínicas, Universidade de São Paulo, São Paulo, SP, Brazil

Dear Editor,

Encephalocraniocutaneous Lipomatosis (ECCL), also known as Haberland Syndrome, is a rare and sporadic genetic condition characterized by congenital alterations that simultaneously affect the skin, eyes, and central nervous system.^1^, ^2^ This association of manifestations in different systems reflects its inclusion in the group of phakomatoses, a heterogeneous set of genetic diseases that share cutaneous, ophthalmological, and neurological anomalies, frequently accompanied by developmental dysplasias and a predisposition to the emergence of tumors.^3^

The pathogenesis is attributed to post-zygotic somatic mutations in genes such as FGFR1, KRAS, and NRAS, which promote aberrant activation of the RAS-MAPK signaling pathway. This process results in mosaicism and ectodermal dysgenesis, explaining the asymmetrical distribution and heterogeneity of clinical manifestations.^2^ Genetic alterations also compromise vasculogenesis, favoring the presence of dysmorphic intracranial vessels, in addition to predisposing to the development of mesenchymal tumors, such as lipomas. One of the most characteristic skin lesions is nevus psiloliparus—a patch of softened alopecia, preferentially located on the scalp, consisting of ectopic mature adipose tissue in the dermis and absence of hair follicles and cutaneous appendages.^2^, ^4^

Due to the sharing of the RAS-MAPK pathway with other RASopathies, such as Neurofibromatosis type 1 (NF1), it is possible to observe some similar cutaneous findings in ECCL, such as café-au-lait spots.^1^, ^2^ This report identified axillary freckles, a finding not yet documented in cases of ECCL, but which can be explained by the same pathogenic mechanism. The diagnosis of ECCL is challenging due to the wide spectrum of clinical presentations and the rarity of the condition, often requiring a high degree of clinical suspicion and a multidisciplinary approach for confirmation.

A five-year-old male patient was referred to the pediatric dermatology clinic with patches of alopecia on the scalp, especially in the vertex and right parietal regions, accompanied by ocular anomalies present since birth. There was also a history of delayed neuropsychomotor development, episodes of seizures, behavioral changes, and a diagnosis of hydrocephalus at three months of age, requiring a ventriculoperitoneal shunt. In addition, the patient was under ophthalmological follow-up, having already undergone ocular surgery for partial excision of epibulbar lipodermoid—a benign congenital ocular choristoma, that is, a malformation consisting of normal tissue in an anomalous location, in this case, ectopic adipose tissue in the bulbar conjunctiva (Fig. 1).

**Fig. 1:**
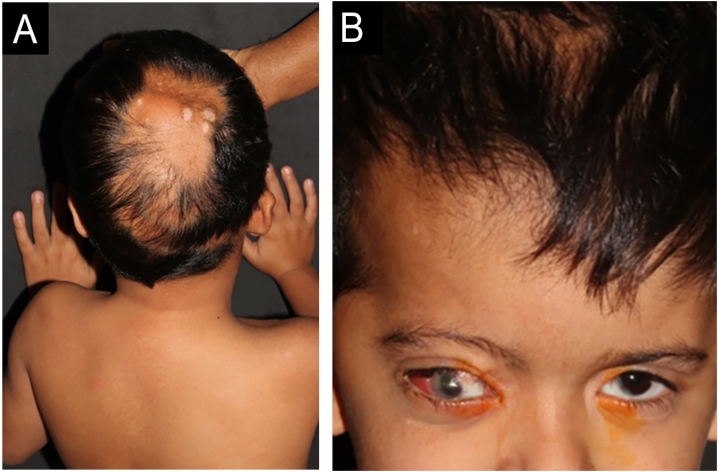
(A) Aplasia cutis over a normochromic alopecia patch in the vertex region; (B) Ipsilateral ocular lipodermoids and low-set right ear.

On physical examination, a heterogeneous alopecic plaque was identified on the scalp, located in the vertex region, with areas of atrophy and others with a softened consistency, similar to adipose tissue, with a clinical appearance compatible with nevus psiloliparus (Fig. 1). The histopathology of one portion of the lesion was suggestive of aplasia cutis. The presence of freckles in the axillary region ipsilateral to the ocular and neurological findings was also observed (Fig. 2). Other anomalies, such as craniosynostosis of the sagittal suture, short stature, low hairline in a "V" shape in the posterior cervical region, low-set right ear with a folded helix, thin lips with eversion of the upper lip, and overlapping fingers, compatible with clinodactyly, were also evidenced. Magnetic resonance imaging (MRI) revealed multiple intracranial lipomas, as well as thickening of the tissue corresponding clinically to the area of ​​alopecia (Fig. 3) in the right frontoparietal region. Based on the clinical picture, histopathological findings, and MRI results, a diagnosis of ECCL was made.

**Fig. 2:**
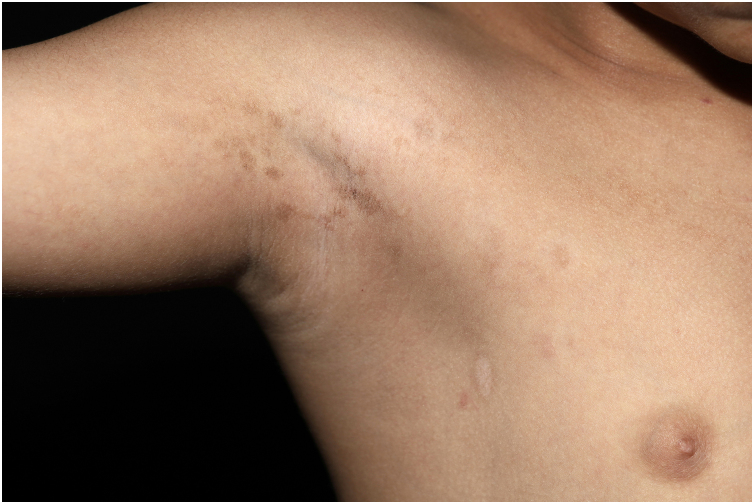
Axillary freckles ipsilateral to ocular and neurological findings.

**Fig. 3:**
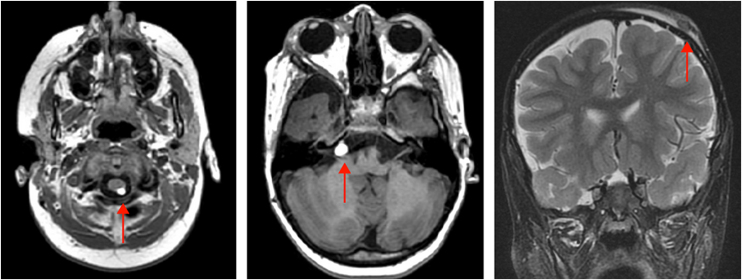
MRI demonstrating a lipoma in the right pontocerebellar cistern and a lipoma posterior to the cervical spinal cord. Subcutaneous tissue thickening is also observed in the right frontoparietal region, clinically corresponding to the alopecia area.

ECCL represents a distinct clinical and genetic neurocutaneous phenotype, characterized by dermatological, ocular, and neurological alterations. Definitive diagnosis of the disease is based on major and minor criteria, distributed among the three affected systems (Table 1).^5^, ^6^ The condition belongs to the group of RASopathies, a set of genetic syndromes caused by mutations in genes that regulate the RAS-MAPK signaling pathway.^2^ These conditions share similar molecular mechanisms and may show overlapping clinical manifestations. Café-au-lait spots, already described in cases of ECCL, are also present in other RASopathies, such as NF1 and cardiofaciocutaneous syndrome.^7^ Additionally, axillary freckles were observed, a manifestation not yet documented in association with ECCL, but which could be explained by the same pathogenic mechanism. This pattern is observed in other RASopathies, such as Neurofibromatosis-Noonan syndrome, NF1, and Legius syndrome, reinforcing the possibility of phenotypic overlap mediated by activation of the RAS-MAPK pathway.^7^, ^8^

**Table 1:** ECCL.

System	Major Criteria	Minor Criteria	Patient Findings
Eyes	1 Choristoma, with or without associated anomalies.	2 Corneal or other anterior chamber anomalies 3 Ocular or palpebral coloboma 4 Calcification of the eyeball	Choristoma (major), coloboma (minor)
Skin	1 Confirmed nevus psiloliparus (NP) 2 Possible NP and ≥ 1 of the minor cutaneous criteria (criteria 2–5) 3 ≥2 minor criteria among criteria 2–5	1 Irregular or banded alopecia, non-scarring 2 Subcutaneous lipoma(s) in the frontotemporal region 3 Focal aplasia/hypoplasia of the skin on the scalp 4 Small nodular lesions on the skin of the eyelids or between the outer corner of the eye and the tragus	Possible NP (major), subcutaneous lipomas in the frontotemporal region, non-scarring alopecia, focal skin aplasia (confirmed by biopsy)
Central Nervous System (CNS)	1 Intracranial lipoma 2 Intraspinal lipoma 3 ≥2 minor criteria	1 Abnormal intracranial vessels 2 Arachnoid cyst or other meningeal abnormality 3 Complete or partial atrophy of a cerebral hemisphere 4 Porencephalic cyst 5 Asymmetrically dilated ventricles or hydrocephalus 6 Calcifications (except in the basal ganglia)	Intracranial lipoma (major), volumetric reduction of the right frontal and temporal lobes, diffuse prominence of cerebrospinal fluid spaces on the right side, small arachnoid cyst in the right middle cranial fossa.
Others	1 Jaw tumor (osteoma, odontoma, or ossifying fibroma) 2 Multiple bone cysts 3 Coarctation of the aorta	N/A	N/A

Definitive Case.

1. Involvement of 3 systems, with major criteria in ≥ 2 of them; or.

2. Involvement of 3 systems, with confirmed or possible nevus psiloliparus (NP) and ≥ 1 of the minor cutaneous criteria (criteria 2–5); or.

3. Involvement of 2 systems with major criteria, one of which must include confirmed or possible nevus psiloliparus and ≥ 1 of the minor cutaneous criteria (criteria 2–5).

Differential diagnoses include Oculocerebrocutaneous Syndrome (Delleman Syndrome), Proteus, Epidermal Nevus and Schimmelpenning Syndrome.^7^, ^9^, ^10^ The therapeutic strategy depends on the symptoms. Endoscopic ventriculostomy of the third ventricle is generally preferred for relief of intracranial hypertension in cases of hydrocephalus.^11^ Surgery is recommended for symptomatic spinal lipomas, aiming to preserve neurological function, with follow-up being essential to monitor the progression of lipomatous lesions and prevent vertebral deformities.^11^

## ORCID IDs

Julia Maria de Oliveira Neumayer: 0009-0001-9651-1942

Milene Tiburcio Narenti Ferradoza: 0000-0002-5864-7259

Luciana Paula Samorano: 0000-0001-7077-8553

Maria Cecilia Rivitti-Machado: 0000-0003-2910-7330

Zilda Najjar Prado de Oliveira: 0000-0002-8596-1999

## Authors' contributions

Ana Clara Maia Palhano: Design and planning of the study; collection, analysis, and interpretation of data; drafting and editing of the manuscript; approval of the final version of the manuscript.

Julia Maria de Oliveira Neumayer: Analysis and interpretation of data; drafting and editing of the manuscript; approval of the final version of the manuscript.

Milene Tiburcio Narenti Ferradoza: Analysis and interpretation of data; drafting and editing of the manuscript; approval of the final version of the manuscript.

Luciana Paula Samorano: Clinical intervention in the case; critical review of the manuscript; approval of the final version of the manuscript.

Maria Cecilia Rivitti-Machado: Clinical intervention in the case; critical review of the manuscript; approval of the final version of the manuscript.

Zilda Najjar Prado de Oliveira: Research orientation; critical review of the manuscript; approval of the final version of the manuscript.

## Ethical approval and informed consent

The study was conducted in accordance with the institution's ethical standards, and informed consent was obtained from the patient's legal guardian.

## Approval statements

All authors read and approved the final version of the manuscript.

## Consent for publication

Written informed consent was obtained from the patient's legal guardian for the publication of this case report and corresponding images.

## Financial support

None declared.

## Research data availability

Not applicable.

## Conflicts of interest

None declared.
